# Establishment and Characterization of an Irinotecan-Resistant Human Colon Cancer Cell Line

**DOI:** 10.3389/fonc.2020.624954

**Published:** 2021-02-22

**Authors:** Zhuo-Xun Wu, Yuqi Yang, Leli Zeng, Harsh Patel, Letao Bo, Lusheng Lin, Zhe-Sheng Chen

**Affiliations:** ^1^Department of Pharmaceutical Sciences, College of Pharmacy and Health Sciences, St. John’s University, New York, NY, United States; ^2^Precision Medicine Center, The Seventh Affiliated Hospital, Sun Yat-Sen University, Shenzhen, China; ^3^Cell Research Center, Shenzhen Bolun Institute of Biotechnology, Shenzhen, China

**Keywords:** chemotherapy, irinotecan, colorectal cancer, multidrug resistance, ABCG2

## Abstract

Colorectal cancer (CRC) is a leading cause of cancer-related deaths worldwide. Irinotecan is widely used as a chemotherapeutic drug to treat CRC. However, the mechanisms of acquired resistance to irinotecan in CRC remain inconclusive. In the present study, we established a novel irinotecan-resistant human colon cell line to investigate the underlying mechanism(s) of irinotecan resistance, particularly the overexpression of ABC transporters. The irinotecan-resistant S1-IR20 cell line was established by exposing irinotecan to human S1 colon cancer cells. MTT cytotoxicity assay was carried out to determine the drug resistance profile of S1-IR20 cells. The drug-resistant cells showed about 47-fold resistance to irinotecan and cross-resistance to ABCG2 substrates in comparison with S1 cells. By Western blot analysis, S1-IR20 cells showed significant increase of ABCG2, but not ABCB1 or ABCC1 in protein expression level as compared to that of parental S1 cells. The immunofluorescence assay showed that the overexpressed ABCG2 transporter is localized on the cell membrane of S1-IR20 cells, suggesting an active efflux function of the ABCG2 transporter. This finding was further confirmed by reversal studies that inhibiting efflux function of ABCG2 was able to completely abolish drug resistance to irinotecan as well as other ABCG2 substrates in S1-IR20 cells. In conclusion, our work established an *in vitro* model of irinotecan resistance in CRC and suggested ABCG2 overexpression as one of the underlying mechanisms of acquired resistance to irinotecan. This novel resistant cell line may enable future studies to overcome drug resistance *in vitro* and improve CRC treatment *in vivo*.

## Introduction

Colorectal cancer (CRC), one of the most common type of malignant tumors, is the third leading cause of cancer-related deaths in the United States and fourth most deadly cancer worldwide (1). Currently, the mainstream therapeutic approaches to treat metastatic CRC are surgery, chemotherapy, irradiation, and targeted therapy (2). The first-line chemotherapy strategy is usually a combination of 5-fluorouracil, leucovorin, and either irinotecan or oxaliplatin (3). The combination chemotherapy regimens have been shown to achieve greater efficacy as compared to single agent treatment (4). Irinotecan, derived from camptothecin, is a broad-spectrum chemotherapeutic agent targeting DNA topoisomerase I (topo I) (5). Topoisomerases are nuclear enzymes that are actively involved in the control of DNA topology during the replication and translation processes (6). It is well established that topo I levels are upregulated in some cancer types, and inhibiting topo I activity can affect the proliferation of tumor cells (7). Since its approval in 1996 by the United States Food and Drug Administration, irinotecan has been the first-line option in treating CRC, gastrointestinal cancers, lung cancer, and a variety of other malignancies (8). In the first-line treatment of mCRC, the combination of irinotecan with bolus or 5-fluorouracil/leucovorin can significantly increase patient survival compared with 5-fluorouracil/leucovorin alone, with an acceptable toxic profile (9). Furthermore, irinotecan-based combinations can be extremely diversified, including combination with kinase inhibitors and cell-cycle checkpoint inhibitors (10). Currently, new derivatives of irinotecan are still under development to improve anticancer efficacy and reduce side effects. After two decades of clinical usage, irinotecan remains a must-have drug in CRC treatment (11). However, one major obstacle of chemotherapy is inevitably the development of drug resistance. To date, the mechanism of irinotecan resistance is still inconclusive and requires further investigation. Studies have suggested several possible mechanisms that may lead to irinotecan resistance, including: 1) reduced topo I expression level, 2) mutation in the metabolizing enzyme or structure of topo I, 3) activation of NF-κB which suppresses the cell apoptosis, and 4) reduced intracellular drug accumulation by active drug efflux (12).

Several ATP-binding cassette (ABC) transporters are well-established mediators of multidrug resistance (MDR). Specifically, ABCB1, ABCG2, and ABCC1 are able to confer cancer cell MDR to a wide range of anticancer drugs with different chemical structures and mechanisms of action. These ABC transporters, by utilizing energy derived from ATP hydrolysis, extrude substrates from the cytoplasm to the extracellular matrix, resulting in decreased sensitivity of cancer cells to substrate drugs. *In vitro* model has been an ideal method to delineate the potential mechanisms contributing to drug resistance. The ABCB1 and ABCG2 transporters were first discovered from cells resistant to colchicine and mitoxantrone, respectively (13). The docetaxel-resistant cell lines were shown to overexpress ABCB1 transporter (14), and the arsenic trioxide-resistant cell line was shown to overexpress ABCB6 transporter (15). In the past few decades, a broad array of chemotherapeutic drugs as well as tyrosine kinase inhibitors (TKI) were identified as substrates of ABC transporters. The chemotherapeutic drugs paclitaxel, vincristine, and colchicine are substrates of ABCB1, while mitoxantrone, topotecan, and etoposide are substrates of ABCG2 (13, 16). In addition, TKIs such as imatinib (17), gefitinib (18), tivantinib (19), pevonedistat (20) are reported to be substrates of ABC transporters. Studies have resulted in mixed conclusions surrounding the role ABCG2 plays in mediating irinotecan resistance (21–24). Both irinotecan and its active metabolite SN-38 were reported to be substrates of ABCG2 (25). One exploratory analysis study was performed and suggested that the response to irinotecan is highly related to tumor *ABCG2* mRNA expression (26). In a retrospective study, it was found that ABCG2 level serves a predictive role for SN-38 resistance (27). In contrast, studies also suggested that ABCG2 was not a predictor of progression-free survival (PFS) in patients receiving irinotecan treatment and no significant correlation was found between ABCG2 level and treatment response (28, 29). Currently, the investigation of irinotecan and ABCG2 were mostly conducted by correlating ABCG2 expression level with response to irinotecan. However, whether irinotecan treatment can lead to ABC transporters overexpression remain inconclusive and should be further explored.

The present study aimed to facilitate the understanding of resistance mechanism of irinotecan in CRC. To achieve this, a colon cancer cell line resistant to irinotecan was established. It was found that, unlike ABCB1 or ABCC1, ABCG2 was overexpressed in irinotecan-resistant S1-IR20 cells. Furthermore, ABCG2 overexpression in S1-IR20 cells also conferred high levels of cross resistance to other ABCG2 substrates. Inhibiting the efflux function of ABCG2 significantly decreased the drug resistance to ABCG2 substrate drugs in S1-IR20 cells. Our results suggested that overexpression of ABCG2 is a key mediator of acquired resistance to irinotecan in CRC; thus, ABCG2 level may be monitored during irinotecan treatment.

## Materials and Methods

### Chemicals

Chemicals were obtained from Sigma Chemical Co (St. Louis, MO) unless stated otherwise. Chemotherapeutic drugs used in this study were irinotecan, SN-38, mitoxantrone, topotecan, doxorubicin, colchicine, paclitaxel, and oxaliplatin. Ko143 was purchased from Enzo Life Sciences (Farmingdale, NY). Venetoclax was requested from Chemietek (Indianapolis, IN). Stock solutions (10 mM) were reconstituted in DMSO for all the drugs except oxaliplatin, which was dissolved in dimethylformamide.

### Development of Drug-Resistant S1-IR20 Cell Line

Human colon cancer S1 cell line was used as the parental cell line to establish the irinotecan-resistant subline. The parental S1 cells were initially cultured in medium with 0.5 μM of irinotecan for 48 h. Subsequently, the surviving cells were cultured in drug-free medium for 7 days and subjected to the next cycle of drug treatment. After 3–5 cycles of drug treatment, the cells were cultured with increased concentrations of irinotecan, a 50% increase each time. Finally, the resultant cell lines that grew exponentially in the presence of 20 μM of irinotecan were designated as drug-resistant subline and named S1-IR20. Established S1-IR20 cells were maintained in drug-free medium for 14 days before experiment.

### Cell Lines and Cell Culture

The human epidermoid carcinoma cell line KB-3-1 and its ABCB1-overexpressing subline KB-C2, ABCC1-overexpressing subline KB-CV60, human NSCLC NCI-H460 and its ABCG2-overexpressing subline NCI-H460/MX20, and human colon cancer cell line S1 and its irinotecan-resistant subline S1-IR20 were maintained in DMEM with 10% FBS. In addition, KB-C2 cells were maintained in the presence of 2 μg/ml of colchicine (30). NCI-H460/MX20 cells were maintained in the presence of 20 nM of mitoxantrone (31). KB-CV60 cells were maintained in the presence of 1 µg/ml of cepharanthine and 60 ng/ml of vincristine (32). All cell lines were maintained in a humid incubator (37°C, 5% CO_2_) and subcultured at 80% confluency.

### Evaluation of S1-IR20 Drug Resistance Profile by MTT Assay

The MTT assay was used to determine the cytotoxicity of chemotherapeutic drugs as previously described (33). Cell viability curves were plotted as percentages relative to untreated controls. The half maximal inhibitory concentrations (IC_50_) were calculated from the curves using GraphPad Prism 7.

### Western Blotting

Protein extraction and visualization were performed as previously described (19). The ABCG2-overexpressing NCI-H460/MX20, ABCB1-overexpressing KB-C2, and ABCC1-overexpressing KB-CV60 protein samples were used as positive control of ABCG2, ABCB1, and ABCC1, respectively. Immunoblotting was carried out with the following antibodies: anti-ABCG2, anti-ABCB1, anti-ABCC1, anti-topo I and anti-GAPDH (catalog number MAB4146, MA1-26528, MA5-16079, 44321M, MA5-15738, Thermo Fisher Scientific Inc., Waltham, MA) and anti-rabbit or anti-mouse secondary HRP-linked antibodies (catalog number 7074S and 7076S, 1:1,000 dilution, Cell Signaling Technology Inc., Danvers, MA). GAPDH was used as a loading control. The protein bands were visualized using an enhanced ECL Kit (Thermo Fisher Scientific Inc., Waltham, MA).

### Immunofluorescence Assay

The localization of ABCG2 transporter was visualized by immunofluorescent microscopy. Both S1 and S1-IR20 cells were seeded in 24-well plates (1 × 10^6^ cells per well). The immunofluorescence experiment was carried out as previously described (34). The antibodies used in this experiment were anti-ABCG2 (1:1,000, Thermo Fisher Scientific Inc., Waltham, MA) and Alexa Fluor 488 conjugated anti-mouse IgG antibody (1:1,000, Thermo Fisher Scientific Inc., Waltham, MA). The nuclei were counterstained with DAPI solution. The immunoreactivity was visualized using a Nikon TE-2000S fluorescence microscope (Nikon Instruments Inc., Melville, NY).

### Intracellular Accumulation of Irinotecan Determined by HPLC Assay

The HPLC assay was carried out with modified protocol as previously described (35, 36). Briefly, both S1 and S1-IR20 cells were seeded in six-well plates (1 × 10^6^ cells per well) and incubated overnight. On the following day, cells were pre-treated with or without Ko143 for 2 h. Subsequently, cells were further incubated with irinotecan in the presence or absence of Ko143 for another 2 h. At the end, cells were harvested and subjected to HPLC analysis.

### Statistical Analysis

All data are presented as the mean ± standard deviation from three independent experiments. Comparisons of differences in the quantitative data among groups were performed using one-way ANOVA. P < 0.05 was considered statistically significant.

## Results

### Establishment of Irinotecan-Resistant Colon Cancer Cell Line and Its Drug Resistance Profile

To obtain the irinotecan-resistant subline, the colon cancer cell line S1 was exposed to increasing concentrations of irinotecan. The resistant subline S1-IR20 was obtained from the population growing in 20 μM of irinotecan. MTT assay was carried out to evaluate the drug resistance profile of S1-IR20 cells. Drug sensitivity of both parental S1 cells and drug-resistant S1-IR20 cells toward a panel of chemotherapeutic drugs is presented in **Table 1**. S1-IR20 showed no significant resistance to ABCB1 substrate drugs paclitaxel (IC_50_ = 0.459 and 0.624 μM for S1 and S1-IR20 lines, respectively), colchicine (IC_50_ = 0.227 and 0.321 μM, respectively), or non-substrate drug oxaliplatin (IC_50_ = 13.62 and 12.61 μM, respectively). In contrast, the resistant cells exhibited high resistance to irinotecan compared to the parental cells (IC_50_ = 0.668 and 31.78 μM for S1 and S1-IR20 lines, respectively). The S1-IR20 cells also showed cross-resistance to SN-38 (47.18-fold), mitoxantrone (37.14-fold), topotecan (41.06-fold), and doxorubicin (18.10-fold) as compared to the parental cells. Therefore, the MTT results suggested that irinotecan-selected S1-IR20 cells may harbor the MDR phenotype.

**Table 1:** The cytotoxicity of chemotherapeutic drugs in S1 and S1-IR20 cell lines.

Drugs	Resistance mechanism	IC_50_ value ± SD^a^ (µM)		Resistance-fold^b^
		S1	S1-IR20	
**Irinotecan**	ABCG2	0.668 ± 0.157	31.78 ± 4.726	47.57^*^
**SN-38**	ABCG2	0.479 ± 0.039	22.60 ± 1.177	47.18^*^
**Topotecan**	ABCG2	0.679 ± 0.073	27.88 ± 3.087	41.06^*^
**Mitoxantrone**	ABCG2	0.070 ± 0.023	5.128 ± 1.126	37.14^*^
**Doxorubicin**	ABCG2, ABCB1	0.329 ± 0.054	5.955 ± 1.146	18.10^*^
**Oxaliplatin**	Non-ABC related	13.62 ± 2.228	12.61 ± 1.262	0.93
**Paclitaxel**	ABCB1, ABCC10	0.459 ± 0.084	0.624 ± 0.158	1.35
**Colchicine**	ABCB1	0.277 ± 0.041	0.321 ± 0.028	1.16

a IC_50_ values are represented as mean ± SD of at least three independent experiments performed in triplicate.

b Resistance fold was calculated by dividing the IC_50_ values of the resistant S1-IR20 cells by the IC_50_ of parental S1 cells.

*P < 0.05 versus the control group.

### Western Blot Analysis

**Figure 1:**
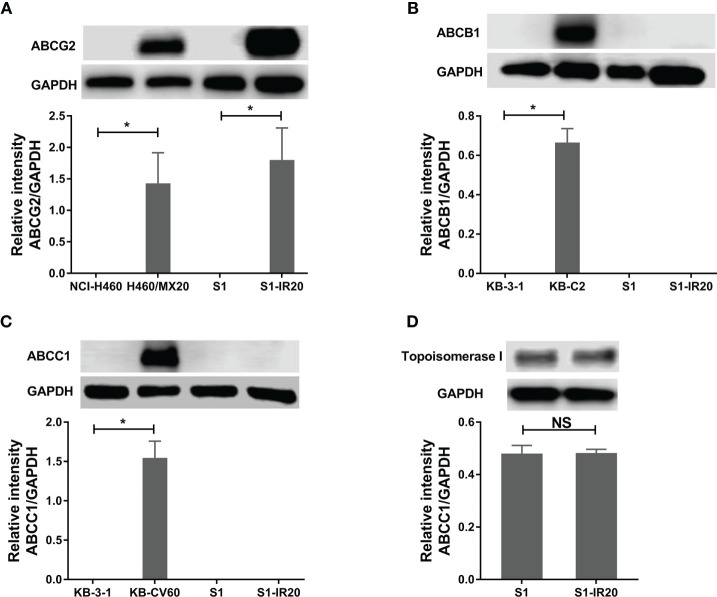
Protein expression profile of S1 and S1-IR20 cells. **(A)** The expression level of ABCG2 in S1 and S1-IR20 cells. The parental NCI-H460 and its ABCG2-overexpressing subline NCI-H460/MX20 were used as negative and positive control of ABCG2, respectively. **(B)** The expression level of ABCB1 in S1 and S1-IR20 cells. The parental KB-3-1 and its ABCB1-overexpressing subline KB-C2 were used as negative and positive control of ABCB1, respectively. **(C)** The expression level of ABCC1 in S1 and S1-IR20 cells. The parental KB-3-1 and its ABCC1-overexpressing subline KB-CV60 were used as negative and positive control of ABCC1, respectively. **(D)** The expression level of topoisomerase I in S1 and S1-IR20 cells. Data are expressed as mean ± SD derived from three independent experiments. *p < 0.05 *versus* the corresponding parental cells. NS, no significant.

Since some ABC transporters have been well established to mediate MDR to a broad range of chemotherapeutic drugs, the expression level of ABCB1, ABCC1, and ABCG2 was evaluated by Western blotting (**Figure 1**). ABCG2 was overexpressed in S1-IR20 cells compared to the parental S1 cells, and the expression level was similar to the ABCG2-overexpressing NCI-H460/MX20 cells (**Figure 1A**). In contrast, both parental S1 and drug-resistant cells displayed no detectable levels of ABCB1 or ABCC1 as shown in **Figures 1B, C**. Since irinotecan’s anticancer activity is by targeting topo I, we assessed the expression level of topo I in S1 and S1-IR20 cells. As shown in **Figure 1D**, topo I was expressed at the same level in both parental and resistant cells. Therefore, the high resistance to irinotecan as well as to mitoxantrone, topotecan, and doxorubicin in S1-IR20 cells may be associated with the overexpression of ABCG2.

### Immunofluorescence Assay

**Figure 2:**
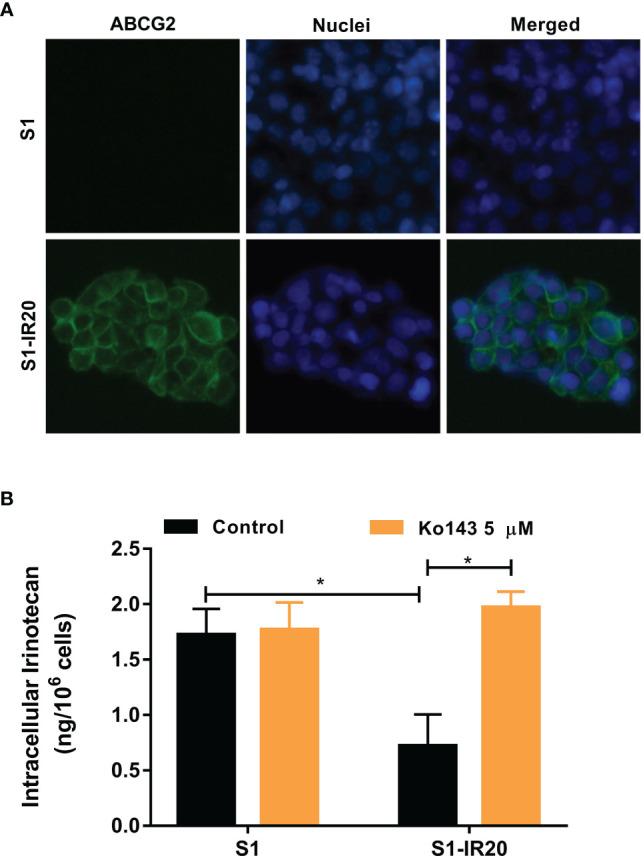
The localization of ABCG2 in S1-IR20 cells and intracellular accumulation of irinotecan in S1 and S1-IR20 cells. **(A)** Cellular membrane localization of the ABCG2 transporter in S1 and S1-IR20 cells. **(B)** The intracellular accumulation of irinotecan was determined in S1 and S1-IR20 cells *via* HPLC assay. Data are expressed as mean ± SD derived from three independent experiments. *p < 0.05 *versus* the control groups.

As a membrane transporter, ABCG2 requires membrane localization to exert its drug efflux function. Therefore, immunofluorescence assay was performed to confirm the overexpression of ABCG2 and visualize its localization in S1-IR20 cells (**Figure 2A**). In parental S1 cells, no detectable green fluorescence was observed, as S1 cells do not overexpress ABCG2. In contrast, strong green fluorescence was observed on the cell membrane of S1-IR20 cells, suggesting that the overexpressed ABCG2 transporter is localized on the cell membrane.

### Intracellular Accumulation of Irinotecan in S1 and S1-IR20 Cells

To evaluate the efflux activity of the overexpressed ABCG2 transporter, we measured the intracellular accumulation of irinotecan in parental S1 and resistant S1-IR20 cells. If irinotecan is exported by the efflux function of the ABCG2 transporter, the intracellular accumulation will be lower in the drug-resistant cells than in the parental cells. As shown in **Figure 2B**, the S1-IR20 cells demonstrated a marked decrease of intracellular accumulation of irinotecan, which is 60% lower than that in the parental S1 cells. Furthermore, the intracellular accumulation of irinotecan in S1-IR20 cells was restored to the same level as S1 cells with the co-treatment of ABCG2 inhibitor Ko143.

### Drug Resistance Phenotype of S1-IR20 Cells Is Abolished by ABCG2 Inhibitor

**Figure 3:**
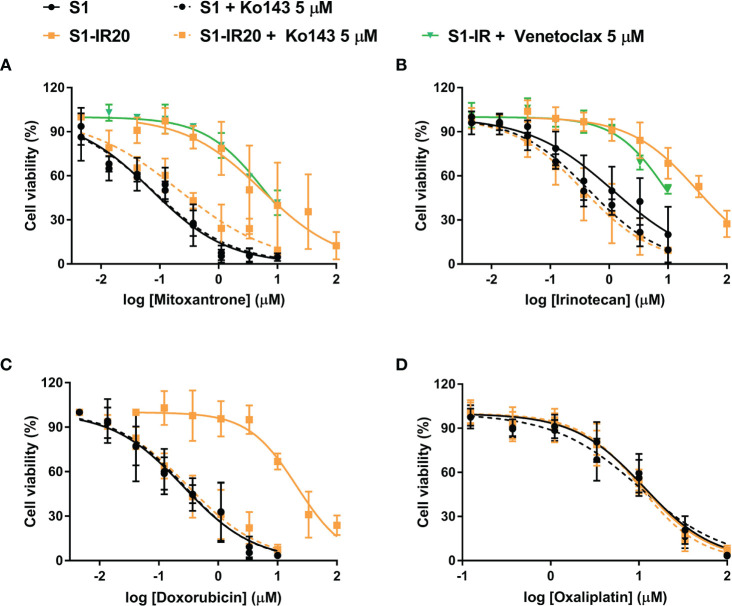
Reversal of ABCG2-mediated MDR using Ko143 in S1 and S1-IR20 cells. The effect of Ko143 and venetoclax on the cytotoxicity (i.e., cell viability curves) of **(A)** mitoxantrone, **(B)** irinotecan, **(C)** doxorubicin, and **(D)** oxaliplatin in S1 and S1-IR20 cells. Data are expressed as mean ± SD derived from three independent experiments.

To confirm that overexpression of ABCG2 is the major factor contributing to the resistance phenotype of S1-IR20 cells, we performed ABCG2-inhibition experiments using the MTT assay. The selective ABCG2 inhibitor Ko143 was used to inhibit the efflux function of ABCG2. As shown in **Figure 3**, resistance to irinotecan as well as to other ABCG2 substrate drugs was abolished by Ko143 in S1-IR20 cells, indicated by the overlapped cell viability curves with the parental cells. In the parental cells, the cell viability curves showed no significant difference in the presence or absence of Ko143. Notably, the cytotoxicity of non-substrate drug oxaliplatin was not altered by Ko143 in both parental and resistant cells. Therefore, these results further confirmed that ABCG2 is the major mediator of drug resistance in S1-IR20 cells. To determine whether S1-IR cells overexpress wild-type or mutant-type ABCG2, we performed reversal studies using venetoclax, an inhibitor of wild-type ABCG2. In S1-IR cells, venetoclax showed no effect to the cytotoxicity of mitoxantrone or irinotecan, suggesting that S1-IR cells may overexpress mutant-type ABCG2.

## Discussion

Chemotherapy is the mainstream strategy for the treatment of CRC, and irinotecan is commonly used as part of the treatment strategies in treating CRC (37). However, chemotherapy inevitably leads to drug resistance, which decreases the anticancer efficacy of chemotherapeutic agents. Furthermore, chemotherapy resistance is often associated with MDR (38). Hence, great effort has been devoted to exploring the mechanisms of MDR in cancer, mostly by establishing drug-selected, acquired drug-resistant cell lines (30, 31). By studying the parental drug-sensitive cells to the drug-resistant cells by molecular biology and cellular methods, several MDR-associated molecules have been revealed (39). Numerous studies have suggested that ABCB1, ABCG2, and ABCC1 are key mediators of MDR through their drug efflux activity (40). In particular, ABCG2 has been reported to impact the pharmacokinetics of irinotecan (41). In contrast, the role of ABCG2 in predicting clinical response of or resistance to irinotecan in CRC is uncertain (22). To date, there are few studies describing the drug resistance mechanisms, e.g., overexpression of efflux pumps in irinotecan-selected resistant human CRC cell line (42, 43). In addition, whether irinotecan treatment will cause ABC transporter overexpression and lead to MDR is unknown. Previous study has established a mitoxantrone-selected drug-resistant S1-M1-80 subline from human S1 colon cancer cell line and suggested ABCG2 overexpression as the major resistance mechanism in the resistant subline (44). However, since mitoxantrone is not mainly used for CRC treatment, establishing an irinotecan-resistant subline would be more suitable to reveal the underlying drug resistance mechanisms and develop effective strategies to overcome the drug resistance. Although *in vivo* drug resistance mechanisms are more complicated than *in vitro*, investigating such *in vitro* drug resistance mechanisms can firstly provide strategies to circumvent or overcome acquired drug resistance in CRC.

In the present study, we established the irinotecan-resistant human colon cancer cell line (S1-IR20) through *in vitro* selection after exposing parental S1 human colon cancer cells to stepwise increasing concentrations of irinotecan. The characterization of the resistant subline S1-IR20 was performed by comparing it to the parental S1 cells. MTT assay was performed to evaluate the cytotoxic effect of irinotecan in parental S1 cells and drug-resistant S1-IR20 cells. This resistant subline exhibited over 40-fold resistance to irinotecan as compared with parental cells, and the drug resistance was stable after at least 2 months in the absence of irinotecan maintenance. Since ABCB1, ABCG2, and ABCC1 are major mediators of MDR, we explored whether these ABC transporters are involved in this irinotecan resistance. Subsequently, the cytotoxicity of a panel of chemotherapeutic drugs were assessed in S1 and S1-IR20 cells. Interestingly, the S1-IR20 cells showed significant cross resistance to SN-38 (47-fold), topotecan (41-fold), mitoxantrone (37-fold), doxorubicin (18-fold), which are known substrates of ABCG2 transporter (34, 45). In contrast, S1-IR20 cells did not exhibit any resistance to paclitaxel, colchicine, and oxaliplatin, suggesting that ABCG2 may be a major contributor to the drug resistance phenotype.

We then evaluated the protein expression level of ABCB1, ABCG2, and ABCC1 in both parental S1 cells and drug-resistant S1-IR20 cells. Our results showed that S1 cells did not have detectable expression levels of ABCB1, ABCG2, or ABCC1, which is consistent with previous studies (46). However, overexpression of ABCG2 but not ABCB1 or ABCC1 was observed in S1-IR20 cells. The expression level of ABCG2 in S1-IR20 cells is comparable to that of mitoxantrone-selected ABCG2-overexpressing NCI-H460/MX20 cells, suggesting that acquired resistance to irinotecan may be associated with ABCG2 overexpression. This result is in line with the drug resistance profile of S1-IR20 cell line. Furthermore, irinotecan exerted anticancer effects by targeting DNA topo I, therefore alteration of topo I expression may contribute to drug resistance (47). However, this is unlikely since S1 and S1-IR20 cells showed similar expression levels of topo I. This result is consistent with a previous study that demonstrated that SN-38 (active metabolite of irinotecan)-selected resistant cell line showed no change in topo I expression level (25). ABCG2 has been characterized as a transporter causing of MDR in many human cancers by actively transporting chemotherapeutic agents out of cancer cells (48). As irinotecan is reported as a substrate of ABCG2, exposing cancer cells to irinotecan may cause an up-regulation of both ABCG2 mRNA and protein expression level. Our results suggested that in the presence of irinotecan, ABCG2 is induced to export the drug from the cell and to prevent cytotoxicity.

Since ABCG2 transporter requires cell membrane localization in order to function as an efflux pump to extrude its substrates from intracellular space, we conducted an immunofluorescence assay to visualize the localization of ABCG2 in S1-IR20 cells. The results clearly showed that the overexpressed ABCG2 transporter is localized on the cell membrane of S1-IR20 cells, suggesting that ABCG2 can actively pump out substrate drugs from the cells, leading to decreased intracellular drug accumulation and thus decreasing drug efficacy. To further confirm that the irinotecan resistance is caused by overexpression of ABCG2, we performed an HPLC assay to directly measure the intracellular accumulation of irinotecan in parental S1 cells and drug-resistant S1-IR20 cells in the presence or absence of ABCG2 inhibitor Ko143. It was observed that the intracellular amount of irinotecan in S1-IR20 cells was significantly lower than in S1 cells, with more than 60% of the drug being pumped out from S1-IR20 cells. In addition, co-treatment with Ko143 was able to restore the accumulation of irinotecan in S1-IR20 cells to the same level as that in the parental S1 cells. Ko143 demonstrated no effect on irinotecan accumulation in parental S1 cells, as the cell line does not overexpress ABCG2 transporter. This result was also validated by ABCG2-inhibition experiments. By performing an MTT assay, the cytotoxic effect of irinotecan as well as ABCG2 substrates mitoxantrone and doxorubicin was compared in the presence or absence of Ko143. Our results showed that Ko143 was able to abolish drug resistance to irinotecan, mitoxantrone, and doxorubicin. In contrast, Ko143 did not alter the cytotoxicity of oxaliplatin, a non-substrate drug, in S1 or S1-IR20 cells. In addition, venetoclax can reverse wild-type ABCG2-mediated MDR but has no inhibition effect to mutant-type ABCG2 (49). Our results showed that venetoclax did not affect to the cytotoxicity of irinotecan and mitoxantrone in S1-IR cells, suggesting that S1-IR cells may overexpress mutant-type ABCG2. However, the actual mutation site of ABCG2 in S1-IR cells remains to be determined.

In summary, our work revealed that overexpression of ABCG2 may be an important mechanism in acquired resistance to irinotecan in CRC. Although clinical data suggest an inconclusive relationship of irinotecan response with ABCG2 expression level, our finding reemphasized the importance of ABCG2 in the development of irinotecan-resistance. Therefore, tumor ABCG2 level may serve as a predictor for irinotecan resistance during long-term treatment. The establishment of this drug-resistant cell line may facilitate the understanding of other potential mechanisms contributing to irinotecan resistance in CRC and for developing effective strategy to improve therapeutic response in CRC patients.

## Data Availability Statement

The original contributions presented in the study are included in the article/supplementary material. Further inquiries can be directed to the corresponding author.

## Author Contributions

Z-XW, LL, and Z-SC conceptualized the study. Z-XW, YY, LZ, HP, and LB developed the methodology. Z-XW wrote and prepared the original draft. Z-XW, LL, and Z-SC wrote, reviewed, and edited the manuscript. Z-SC supervised the study. All authors contributed to the article and approved the submitted version.

## Funding

This study was partially supported by the Department of Pharmaceutical Sciences, St. John’s University.

## Conflict of Interest

The authors declare that the research was conducted in the absence of any commercial or financial relationships that could be construed as a potential conflict of interest.
